# Supplementary material for: Development of a New Chemiluminescent Enzyme Immunoassay Using a Two-Step Sandwich Method for Measuring Aldosterone Concentrations
Source: Diagnostics (Basel). 2021 Mar 4;11(3):433. doi: 10.3390/diagnostics11030433 (PMC8001557; doi:10.3390/diagnostics11030433)
Supplement: Supplementary file 1 [file diagnostics-11-00433-s001.pdf]

Supplementary Material

Limit of blank, limit of detection, and limit of quantitation for the improved assay

The limit of blank (LoB), limit of detection (LoD), and limit of quantification (LoQ) at coefficient of variation (CV) values of 10% using the improved reagent were 0.5, 1.3, and 3.0 pg/mL, respectively (Table S1 and Fig. S1). These values were calculated by referring to EP17-P of the National Committee for Clinical Laboratory Standards (NCCLS). The low measurement accuracy was improved by the novel CLEIA method, and the LoD changed from 50 to 1.3 pg/mL. In addition, the upper limit of measurement increased from 1600 to 3200 pg/mL.

Repeatability of the improved assay

We measured five types of control and plasma samples, from low to high concentrations, 20 times in a row, and calculated the CVs based on the mean and standard deviation values. The CVs were 1.2–3.0% (Tables S2 and S3).

Intermediate precision of the improved assay

We measured five plasma samples from low to high concentrations for 22 days, and calculated the CVs based on the mean and standard deviation values. The CVs were 1.6–2.7% (Table S4).

Linearity of the improved assay

Three types of serum and plasma samples, from low to high concentrations, were diluted 10 times with diluent solution. Linearity was established via the double-measuring method in the range of 0 to 3569.9 pg/mL (Tables S5 and S6, Figs. S2 and S3).

Recovery rates for the improved assay

The test was performed using base samples (Sample Series A) and aldosterone solutions (Sample series B) at a volume ratio of 9:1. The control samples were prepared similarly by adding specimen diluent to the base samples. The recovery rates were 92.5–107.8% (Table S7).

Interference testing of the improved assay

Interference testing was performed by adding bilirubin F, bilirubin C, hemoglobin, chyle, and rheumatoid factor to pooled plasma samples with low and high concentrations. Bilirubin F was not affected at a concentration of 19.5 mg/dL, bilirubin C was not affected at 19.7 mg/dL, hemoglobin was not affected at 500 mg/dL, chyle was not affected at 1610 FTU, and rheumatoid factor was not affected at 500 IU/mL (Table S8).

Cross-reactivity of the improved assay

The cross-reactivity was assessed by adding a cross-reactive substance to the low-concentration pooled plasma sample and using the following formula:

% Cross-reactivity = 100 × [Concentration of test substance found]/[Concentration of test substance added].

The cross-reactivity rates were low for all substances (Table S9).

Hook effect of the improved assay

Aldosterone was diluted from 10 to 1,000,000 pg/mL with aldosterone-free serum to evaluate the hook effect. The hook effect was not observed at concentrations of up to 1,000,000 pg/mL (Fig S4).

Correlation of aldosterone concentrations between the improved reagent and LC-MS/MS

This analysis was performed using 75 serum samples. The Passing–Bablok regression analysis of the aldosterone concentrations obtained using the new CLEIA assay and LC-MS/MS in a concentration range of up to 3200 pg/mL are shown in Fig S5A. The slope was 0.976, the intercept was 1.1, and 95% confidence intervals were calculated. The Bland–Altman analysis showed that the mean difference in aldosterone between the two assays was −3.5 pg/mL, with a 95% confidence interval of −9.1 to 2.2 pg/mL (Fig S5B).

Correlation of aldosterone concentrations determined with RIA and LC-MS/MS

This analysis was also performed using 75 serum samples. The results of the Passing–Bablok regression analysis between the aldosterone concentrations obtained using RIA and LC-MS/MS are shown in Fig S5C. The slope was 1.134, the intercept was 51.2, and 95% confidence intervals were calculated. The Bland–Altman analysis showed that the mean difference in aldosterone

between the two assays was 96.1 pg/mL, with a 95% confidence interval of 68.8 to 123.5 pg/mL (Fig S5F).

Correlation of aldosterone concentrations measured with the current reagent and LC-MS/MS

This analysis was also performed using 75 serum samples. The results of the Passing–Bablok regression analysis of the aldosterone concentrations obtained using the current CLEIA assay and LC-MS/MS are shown in Fig S5G. The slope was 1.277, the intercept was 36.7, and 95% confidence intervals were calculated. The Bland–Altman analysis showed that the mean difference in aldosterone between the two assays was 90.2 pg/mL, with a 95% confidence interval of 66.8 to 113.6 pg/mL (Fig S5J).

Correlation of aldosterone concentrations measured with the current reagent and RIA

This analysis was also performed using 75 serum samples. The results of the Passing–Bablok regression analysis of the aldosterone concentrations obtained using the current CLEIA assay and RIA are shown in Fig S5K. The slope was 1.141, the intercept was -26.4, and 95% confidence intervals were calculated. The Bland-Altman analysis showed that the mean difference in aldosterone between the two assays was -3.8 pg/mL, with a 95% confidence interval of –21.3 to 13.7 pg/mL (Fig S5N).

Table S1. Limit of blank (LoB), limit of detection (LoD), and limit of quantitation (LoQ) of the aldosterone concentrations for the CLEIA method using the improved reagent.

Study	Concentration (pg/mL)
LoB	0.5
LoD	1.3
LoQ	3.0

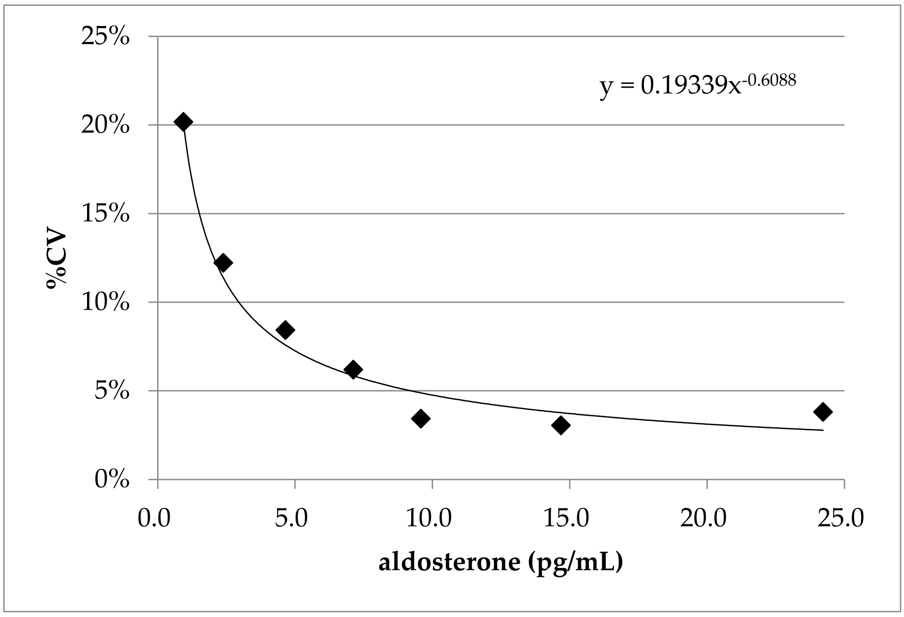

Fig S1. Limit of quantitation of the aldosterone concentrations using the CLEIA method with the improved reagent.

Table S2. Repeatability of the aldosterone measurements in control samples using CLEIA with the improved reagent. Data are presented in pg/mL.

	Control 1	Control 2	Control 3	Control 4	Control 5
N	20	20	20	20	20
Mean	22.4	80.9	600.5	1623.8	2771.6
SD	0.7	2.1	14.7	25.9	43.5
CV	3.0%	2.6%	2.4%	1.6%	1.6%

Table S3. Repeatability of the aldosterone measurements conducted in plasma samples using CLEIA with the improved reagent. Data are presented in pg/mL.

	Plasma 1	Plasma 2	Plasma 3	Plasma 4	Plasma 5
N	20	20	20	20	20
Mean	21.8	83.8	596.0	1535.5	2382.1
SD	0.5	1.3	9.8	18.7	40.6
CV	2.4%	1.5%	1.6%	1.2%	1.7%

Table S4. Intermediate precision of the aldosterone concentrations measured using CLEIA with the improved reagent. Data are presented in pg/mL.

	Day1	Day4	Day8	Day14	Day18	Day22	Mean	SD	CV
Sample1	24.1	24.5	23.0	24.3	24.7	24.2	24.1	0.66	2.7%
Sample2	75.5	78.7	76.0	75.8	76.4	77.3	76.5	1.28	1.7%
Sample3	568.9	579.8	569.2	592.1	568.4	582.2	575.7	10.34	1.8%
Sample4	1527.1	1595.2	1504.3	1483.9	1493.3	1543.9	1520.8	44.62	2.9%
Sample5	2506.1	2477.5	2415.9	2519.5	2500.8	2532.1	2484.0	40.96	1.6%

Table S5. Linearity of aldosterone measured in serum samples using CLEIA with the improved reagent.

Dilution		Serum 1	Serum 2	Serum 3
Aldosterone (pg/mL)	0/10	0.5	0.1	0.4
	1/10	13.5	49.4	266.1
	2/10	26.3	102.0	545.2
	3/10	40.0	152.8	824.7
	4/10	53.7	203.7	1100.3
	5/10	64.2	261.2	1354.2
	6/10	80.9	316.8	1631.8
	7/10	95.4	364.3	1938.1
	8/10	107.6	429.3	2213.1
	9/10	118.9	475.8	2531.0
	10/10	134.0	515.8	2778.5
Recovery rate	1/10	100.7%	95.8%	95.8%
	2/10	98.1%	98.9%	98.1%
	3/10	99.5%	98.7%	98.9%
	4/10	100.2%	98.7%	99.0%
	5/10	95.8%	101.3%	97.5%
	6/10	100.6%	102.4%	97.9%
	7/10	101.7%	100.9%	99.6%
	8/10	100.4%	104.0%	99.6%
	9/10	98.6%	102.5%	101.2%
	10/10	100.0%	100.0%	100.0%

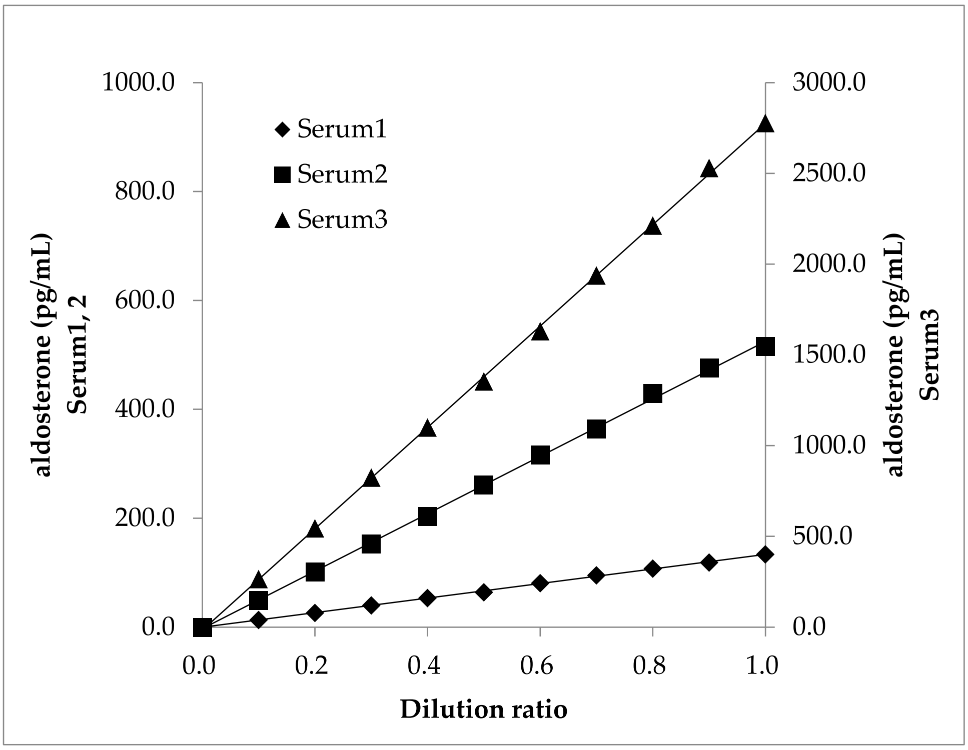

Fig S2. Linearity of aldosterone measured in serum samples using CLEIA with the improved reagent.

Table S6. Linearity of aldosterone in plasma samples measured using CLEIA with the improved reagent.

	Dilution	Plasma 1	Plasma 2	Plasma 3	Plasma 4
Aldosterone (pg/mL)	0/10	0.3	0.4	0.4	0.4
	1/10	14.9	63.5	279.6	375.1
	2/10	29.3	128.5	553.4	727.0
	3/10	42.1	190.7	825.7	1051.8
	4/10	58.4	254.3	1103.5	1386.8
	5/10	76.5	324.7	1385.8	1790.5
	6/10	91.7	394.0	1715.0	2145.8
	7/10	104.0	453.5	1943.7	2497.8
	8/10	121.4	527.0	2266.2	2838.4
	9/10	133.7	582.7	2582.4	3129.2
	10/10	149.0	645.6	2789.9	3569.6
Recovery rate	1/10	100.0%	98.4%	100.2%	105.1%
	2/10	98.3%	99.5%	99.2%	101.8%
	3/10	94.2%	98.5%	98.7%	98.2%
	4/10	98.0%	98.5%	98.9%	97.1%
	5/10	102.7%	100.6%	99.3%	100.3%
	6/10	102.6%	101.7%	102.5%	100.2%
	7/10	99.7%	100.3%	99.5%	100.0%
	8/10	101.8%	102.0%	101.5%	99.4%
	9/10	99.7%	100.3%	102.8%	97.4%
	10/10	100.0%	100.0%	100.0%	100.0%

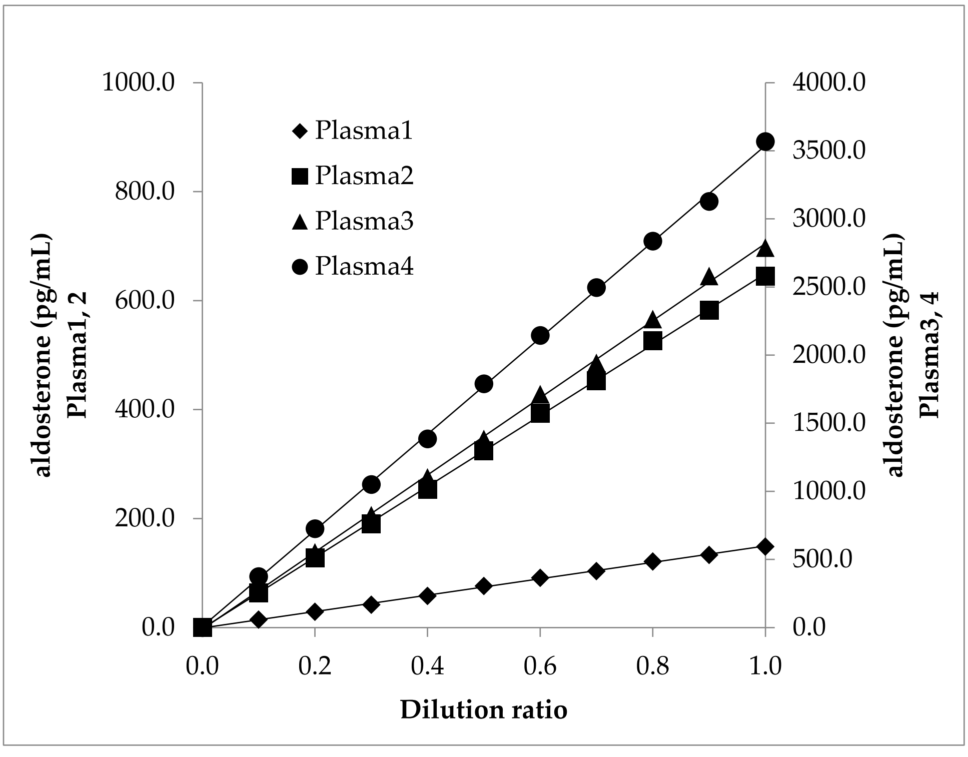

Fig S3. Linearity of aldosterone measured in plasma samples using CLEIA with the improved reagent.

Table S7. Recovery of aldosterone measured using CLEIA with the improved reagent.

	Sample Series A (pg/mL)	Sample Series B (pg/mL)	After Mixed Sample Series (pg/mL)	Obtained Value (pg/mL)	Recovery (%)
Sample1	15.6	200.0	35.4	19.8	99.0%
Sample2	15.6	1600.0	163.8	148.2	92.6%
Sample3	15.6	5000.0	507.9	492.3	98.5%
Sample4	542.4	200.0	560.9	18.5	92.5%
Sample5	542.4	1600.0	696.5	154.1	96.3%
Sample6	542.4	5000.0	1034.8	492.4	98.5%
Sample7	2512.5	1600.0	2665.9	153.4	95.9%
Sample8	2512.5	3200.0	2857.5	345.0	107.8%
Sample9	2512.5	5000.0	3027.2	514.7	102.9%

Table S8. Interference in the aldosterone concentration data obtained using CLEIA with the improved reagent.

Interfering Substance		Plasma 1		Plasma 2	
		Aldosterone Concentrations [pg/mL]	Interference [%]	Aldosterone Concentrations [pg/mL]	Interference [%]
Bilirubin F (mg/dL)	0	71.2	100.0%	480.2	100.0%
	3.9	71.2	100.0%	490.9	102.2%
	7.8	71.0	99.7%	473.0	98.5%
	11.7	71.5	100.4%	481.8	100.3%
	15.6	71.7	100.7%	470.1	97.9%
	19.5	70.6	99.2%	486.0	101.2%
Bilirubin C (mg/dL)	0	67.7	100.0%	470.0	100.0%
	3.9	66.9	98.8%	469.4	99.9%
	7.9	67.6	99.9%	459.5	97.8%
	11.8	68.8	101.6%	462.2	98.3%
	15.8	69.6	102.8%	481.1	102.4%
	19.7	69.3	102.4%	471.4	100.3%
Hemoglobin (mg/dL)	0	67.5	100.0%	475.9	100.0%
	100	69.3	102.7%	472.3	99.2%
	200	69.0	102.2%	474.3	99.7%
	300	67.3	99.7%	465.0	97.7%
	400	67.7	100.3%	467.7	98.3%
	500	67.6	100.1%	481.6	101.2%
Chyle (FTU)	0	67.8	100.0%	477.4	100.0%
	322	68.1	100.4%	476.8	99.9%
	644	67.4	99.4%	470.1	98.5%
	966	67.0	98.8%	465.9	97.6%
	1288	67.4	99.4%	478.9	100.3%
	1610	67.2	99.1%	473.8	99.2%
Rheumatoid factor (IU/mL)	0	73.4	100.0%	517.0	100.0%
	100	73.7	100.4%	500.3	96.8%
	200	72.5	98.8%	522.4	101.0%
	300	72.8	99.2%	503.6	97.4%
	400	73.6	100.3%	510.4	98.7%
	500	72.9	99.3%	516.1	99.8%

Table S9. Cross-reactivity of the aldosterone concentrations obtained using CLEIA with the improved reagent.

Cross-reacting substance	Cross-reactivity	Concentration Tested
Corticosterone	0.00134%	100 μg/mL
18-Hydroxycorticosterone	0.00042%	
Cortisol	<0.00000%	
Cortisone	<0.00000%	
Deoxycorticosterone	0.00006%	
Progesterone	<0.00000%	
Tetrahydrocorticosterone	<0.00000%	
Dexamethasone	<0.00000%	
Prednisolone	<0.00000%	
Spironolactone	<0.00000%	
Eplerenone	<0.00000%	

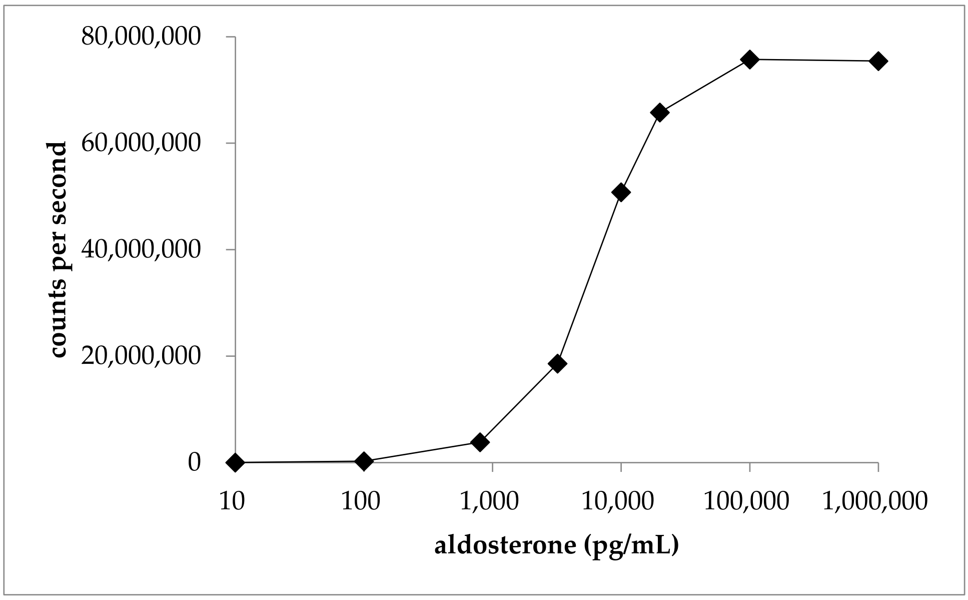

Fig S4. Hook effect of aldosterone using CLEIA with the improved reagent.

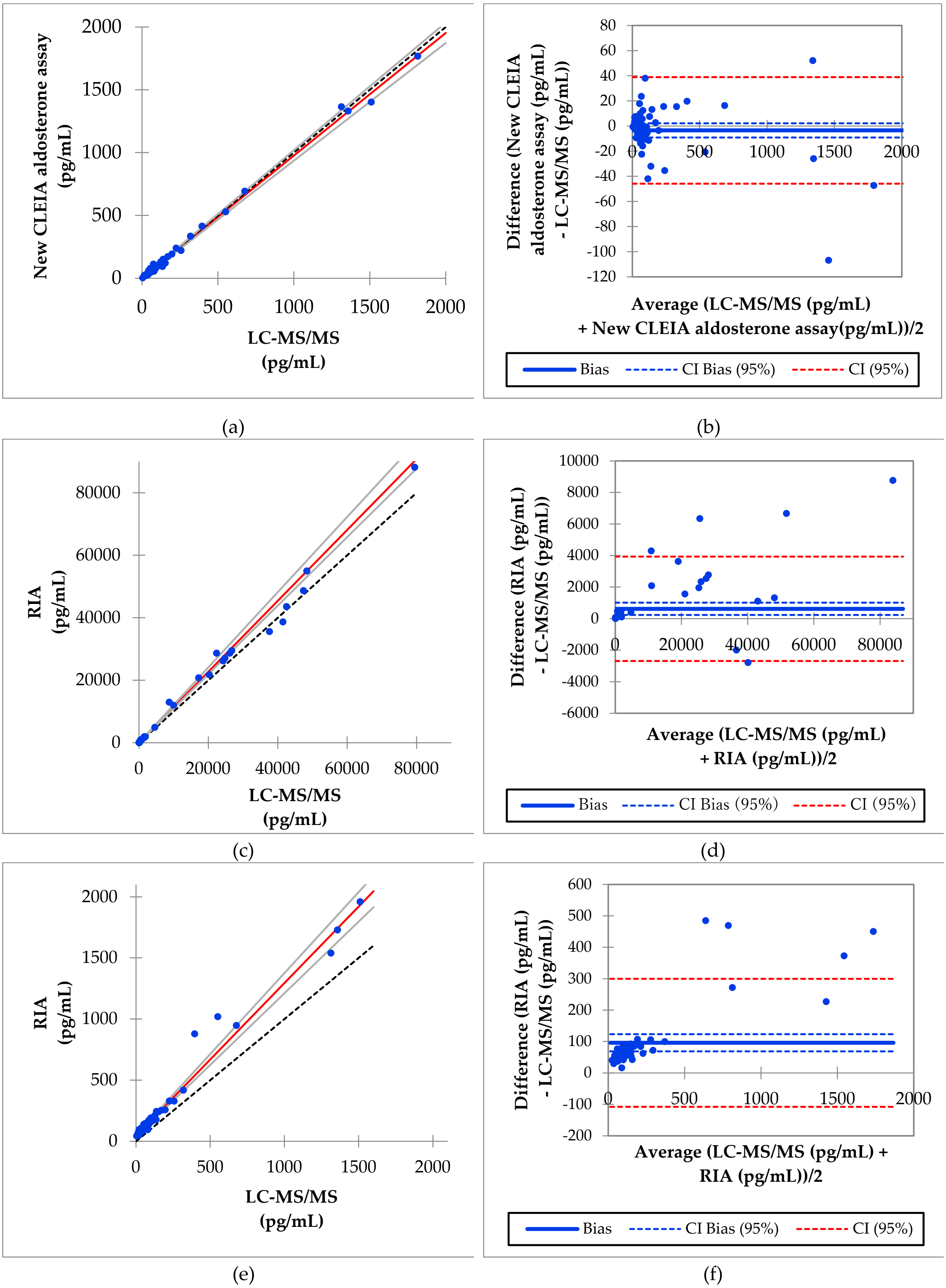
New CLEIA aldosterone assay (pg/mL)
LC-MS/MS (pg/mL)
(a)
Difference (New CLEIA aldosterone assay (pg/mL) - LC-MS/MS (pg/mL))
Average (LC-MS/MS (pg/mL) + New CLEIA aldosterone assay(pg/mL))/2
Bias
CI Bias (95%)
CI (95%)
(b)
RIA (pg/mL)
LC-MS/MS (pg/mL)
(c)
Difference (RIA (pg/mL) - LC-MS/MS (pg/mL))
Average (LC-MS/MS (pg/mL) + RIA (pg/mL))/2
Bias
CI Bias (95%)
CI (95%)
(d)
RIA (pg/mL)
LC-MS/MS (pg/mL)
(e)
Difference (RIA (pg/mL) - LC-MS/MS (pg/mL))
Average (LC-MS/MS (pg/mL) + RIA (pg/mL))/2
Bias
CI Bias (95%)
CI (95%)
(f)

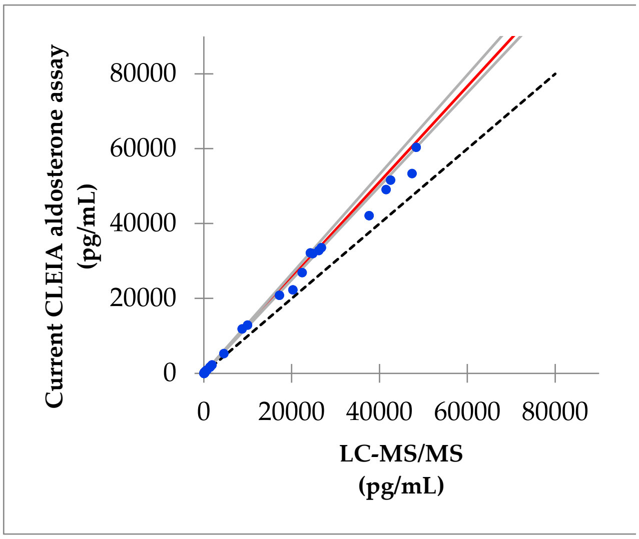
Current CLEIA aldosterone assay (pg/mL)
80000
60000
40000
20000
0
0
20000
40000
60000
80000
LC-MS/MS (pg/mL)

(g)

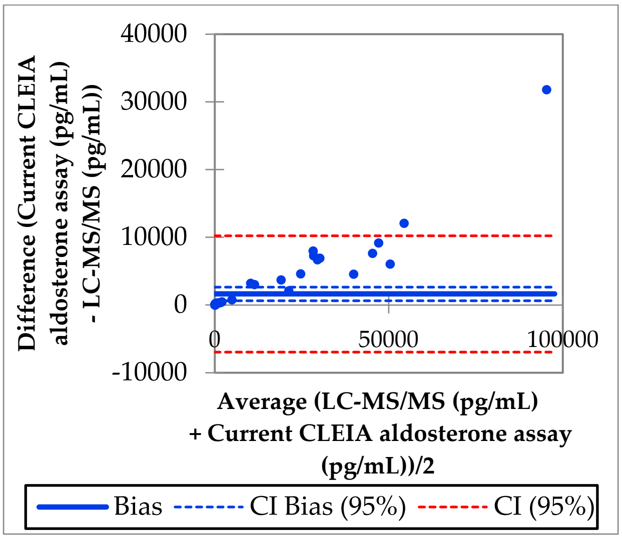
Difference (Current CLEIA aldosterone assay (pg/mL) - LC-MS/MS (pg/mL))
40000
30000
20000
10000
0
-10000
0
50000
100000
Average (LC-MS/MS (pg/mL) + Current CLEIA aldosterone assay (pg/mL))/2
Bias
CI Bias (95%)
CI (95%)

(h)

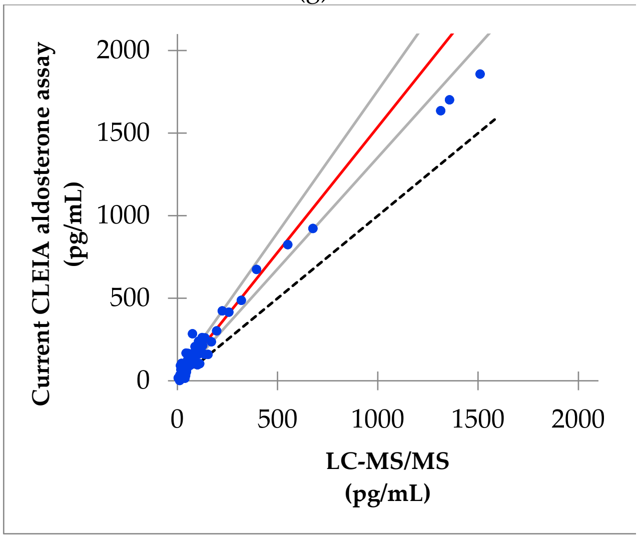
Current CLEIA aldosterone assay (pg/mL)
2000
1500
1000
500
0
0
500
1000
1500
2000
LC-MS/MS (pg/mL)

(i)

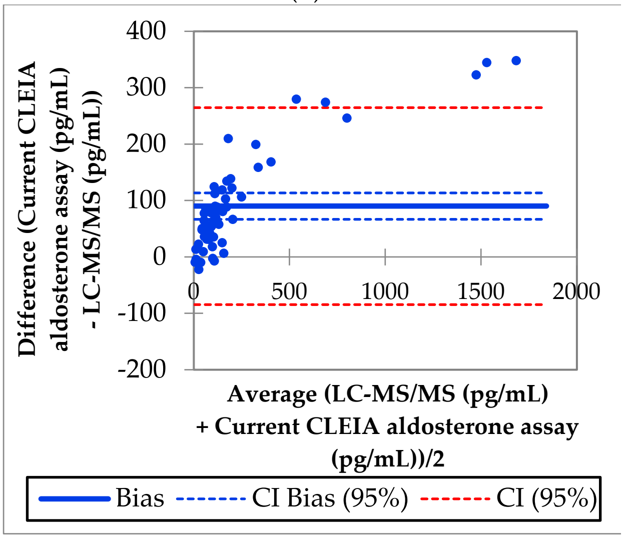
Difference (Current CLEIA aldosterone assay (pg/mL) - LC-MS/MS (pg/mL))
400
300
200
100
0
-100
-200
0
500
1000
1500
2000
Average (LC-MS/MS (pg/mL) + Current CLEIA aldosterone assay (pg/mL))/2
Bias
CI Bias (95%)
CI (95%)

(j)

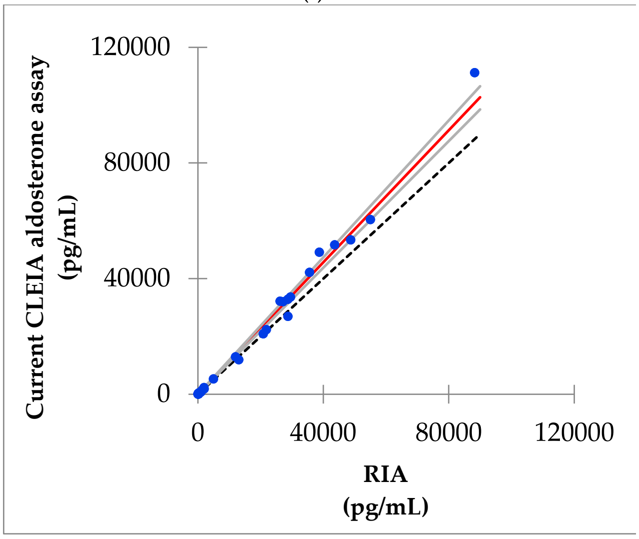
Current CLEIA aldosterone assay (pg/mL)
120000
80000
40000
0
0
40000
80000
120000
RIA (pg/mL)

(k)

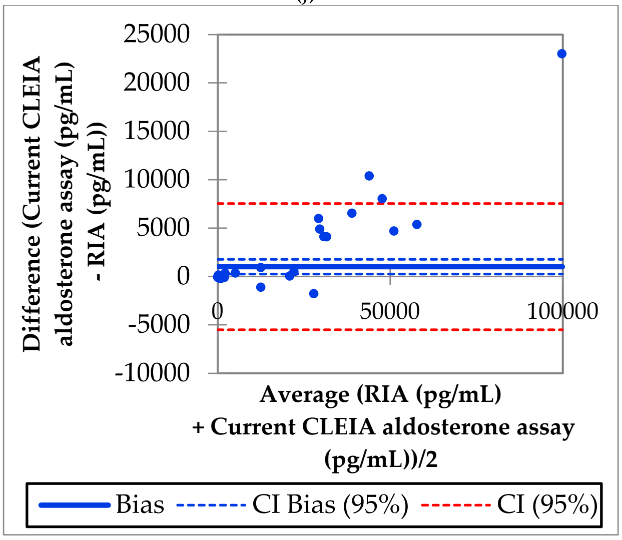
Difference (Current CLEIA aldosterone assay (pg/mL) - RIA (pg/mL))
25000
20000
15000
10000
5000
0
-5000
-10000
0
50000
100000
Average (RIA (pg/mL) + Current CLEIA aldosterone assay (pg/mL))/2
Bias
CI Bias (95%)
CI (95%)

(l)

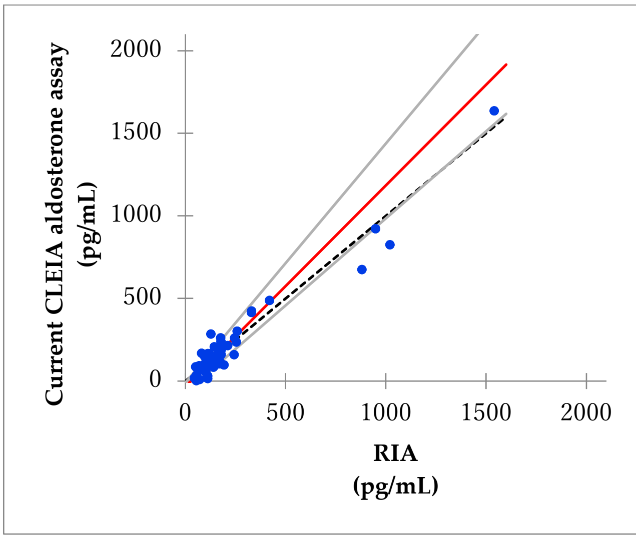

(m)

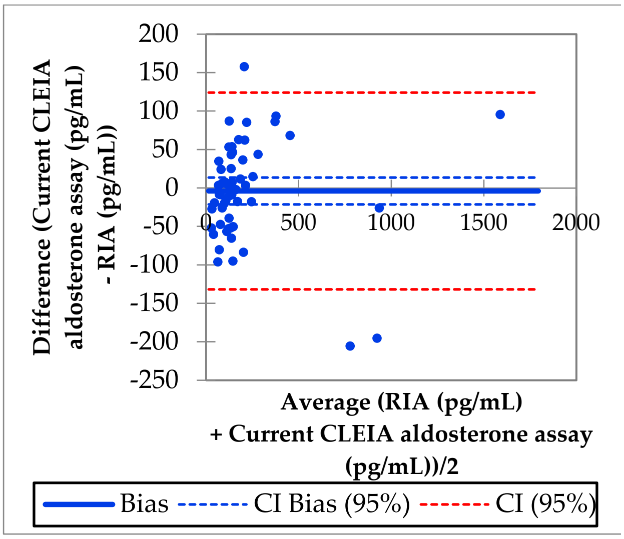

(n)

Fig S5. Results of the Passing–Bablok regression and Bland-Altman analysis.

(a) Passing–Bablok regression of aldosterone concentrations measured using the improved reagent (new CLEIA aldosterone assay) versus LC-MS/MS; (b) Bland-Altman analysis of aldosterone concentrations measured using the improved reagent versus LC-MS/MS; (c) Passing–Bablok regression of aldosterone concentrations measured using the RIA versus LC-MS/MS; (d) Bland-Altman analysis of aldosterone concentrations measured using the RIA versus LC-MS/MS; (e) Passing–Bablok regression of aldosterone concentrations measured using the RIA versus LC-MS/MS (zoom in on the low concentration area); (f) Bland-Altman analysis of aldosterone concentrations measured using the RIA versus LC-MS/MS (zoom in on the low concentration area); (g) Passing–Bablok regression of aldosterone concentrations measured using the current CLEIA aldosterone assay versus LC-MS/MS; (h) Bland-Altman analysis of aldosterone concentrations measured using the current CLEIA aldosterone assay versus LC-MS/MS; (i) Passing–Bablok regression of aldosterone concentrations measured using the current CLEIA aldosterone assay versus LC-MS/MS (zoom in on the low concentration area); (j) Bland-Altman analysis of aldosterone concentrations measured using the current CLEIA aldosterone assay versus LC-MS/MS (zoom in on the low concentration area); (k) Passing–Bablok regression of aldosterone concentrations measured using the current CLEIA aldosterone assay versus the RIA; (l) Bland–Altman analysis of aldosterone concentrations measured using the current CLEIA aldosterone assay versus the RIA; (m) Passing–Bablok regression of aldosterone concentrations measured using the current CLEIA aldosterone assay versus the RIA (zoom in on the low concentration area); (n) Bland–Altman analysis of aldosterone concentrations measured using the current CLEIA aldosterone assay versus the RIA (zoom in on the low concentration area)
